# The Role of Anion Exchanger on Pulmonary Vascular Response to Sustained Alveolar Hypoxia in the Isolated Perfused Rabbit Lung

**Published:** 2015-05

**Authors:** Farzaneh Ketabchi, Somayeh Mansoori, Seyed Mostafa Shid Moosavi

**Affiliations:** Department of Physiology, School of Medicine, Shiraz University of Medical Sciences, Shiraz, Iran

**Keywords:** Hypoxia, Anion exchanger, Lung

## Abstract

**Background:**

Some respiratory diseases may induce alveolar hypoxia thereby hypoxic pulmonary vasoconstriction (HPV). However, the mechanisms of this physiologic phenomenon are not fully understood. This study was the first to investigate the role of anion exchanger in sustained HPV.

**Methods:**

Experiments were performed in the isolated perfused rabbit lung. After preparation, the lungs were divided into six groups: two DIDS (4,4-diisothiocyanostilbene 2,2-disulfonic acid, anion exchanger inhibitor)-treated [200 µM (n=5) or 400 µM (n=3)] hypoxic groups, two HCO_3_^-^ free hypoxic groups, one control hypoxic group (n=7) and one control normoxic group (n=4). DIDS were added to the perfusate at 10 minutes before starting the experiments. In the HCO_3_^-^ free groups, HEPES (4-(2-Hydroxyethyl)piperazine-1-ethanesulfonic acid) were added to the perfusate instead of bicarbonate. Furthermore, in the HEPES1 (n=4) and HEPES2 (n=4) groups, the lungs were ventilated with hypoxic gas with or without CO_2_, respectively.

**Results:**

Ventilation of the lungs with hypoxic gas resulted in biphasic HPV, the acute (0-20 minutes) and sustained (20-60 minutes) phases. No alteration in both phases of HPV was detected by DIDS (200 µM). However, DIDS (400 µM), extended the ascending part of acute HPV until min 24. Both phases of HPV were decreased in the HEPES1 group. However, in the HEPES 2 group, HPV tended to increase during the rising part of the acute phase of HPV.

**Conclusions:**

Since DIDS (400 µM) extended acute phase of HPV, and HCO_3_^-^ free perfusate buffer enhanced rising phase of it, therefore it can be suggested that anion exchanger may modulate HPV especially during the acute phase.

The abstract of this article was presented as a poster in the congress of European Respiratory Society (ERS) on Monday, 08 September 2014, Munich, Germany and was published in the ERJ September 1, 2014 vol. 44 no. Suppl 58 P2343.

## Introduction

Millions of people suffer every day from acute and chronic respiratory diseases.^1-3^ Meanwhile, chronic obstructive pulmonary disease and asthma include the major respiratory diseases worldwide and hypoventilation caused by them may induce local or global alveolar hypoxia. Furthermore, in high altitude, alveolar oxygen pressure is reduced following a decrease in barometric pressure. It has been revealed that pulmonary vessels, despite systemic vessels, are constricted during exposure to hypoxic gas. This physiologic response is identified as hypoxic pulmonary vasoconstriction (HPV) which could locally regulate pulmonary blood flow distribution without any significant alteration in pulmonary artery pressure. However, chronic and global alveolar hypoxia may lead to pulmonary hypertension, which could seriously influence heart and lung functions.^4-7^ Although alveolar hypoxia induces monophasic pulmonary vasoconstriction in vivo, it causes biphasic vasoconstriction in the isolated perfused lung and isolated pulmonary artery, the acute and sustained phases.^8-11^ However, the mechanisms involved in HPV are still controversial.^12^^,^^13^

HPV is reported to be higher in the presence of HCO_3_^-^ perfusate buffer compared with HCO_3_^-^ free perfusate buffer.^14^ Correcting the pH by HCO_3_^-^ during hypercapnia increases pulmonary vascular tone and substitution of HCO_3_^-^ by HEPES inhibits it.^15^ The combination of hypoxia and hypercapnia (pH corrected by HCO_3_^-^) also enhances pulmonary vascular resistance during the acute phase of HPV.^8^^,^^16^

These data suggest the possible role for extracellular HCO_3_^-^ on the regulation of pulmonary vascular tone. Extracellular HCO_3_^-^ is suggested to enter the pulmonary artery smooth muscle cell via two types of Cl-HCO_3_^-^ exchanger Na-dependent and Na-independent.^17^^,^^18^ Administration of DIDS (4,4-diisothiocyanostilbene 2,2-disulfonic acid, anion exchanger inhibitor), a potent and relatively specific inhibitor of anion exchanger or acetazolamide, a carbonic anhydrase inhibitor or induction the Na-free medium decrease pulmonary vascular tone during hypercapnia in the isolated pulmonary artery.^15^ It is also reported that Na-dependent anion exchanger activity and intracellular pH are increased during hypoxia in the rat pulmonary smooth muscle cells.^18^ Nevertheless, the exact role of HCO_3_^- ^and anion exchanger in HPV has not been addressed yet. Considering the above, the present study attempts to investigate the effect of two concentrations of anion exchanger inhibitor^19^^,^^20^ on sustained HPV. In some experiments, HCO_3_^-^ was replaced by HEPES (4-(2-Hydroxyethyl)piperazine-1-ethanesulfonic acid) to evaluate the role of extracellular HCO_3_^-^ on HPV.

## Materials and Methods

*Lung Isolation, Perfusion, and Ventilation*

Animal experiments were approved by the Center for Experimental Animal Laboratory and the Ethical Committee of Animal Care at Shiraz University of Medical Sciences (Shiraz, Iran).

The model of isolated perfused rabbit lung has been described previously.^21^^,^^22^ Briefly, 27 white New Zealand male rabbits (body weight 1.96±0.05 kg) were deeply anaesthetized with continues injection of ketamine (30-50 mg/kg) and xylazine (6-10 mg/kg) to marginal ear vein and heparinized (1500 U/kg body weight). The trachea was cannulated and animals were ventilated (tidal volume 17.94±0.44 ml, respiratory rate 30/min). Chest wall was opened and about 40 ml heparinized blood was collected by cardiac puncture. Blood was centrifuged at 4ºC at 4000×g for 7 minutes and about 20 ml of plasma was stored in ice. Then, pulmonary artery was cannulated and the lung perfused with 4ºC air bubble-free Krebs-Henseleit solution (perfusate) through the pulmonary artery cannula connected to a peristaltic pump with a flow rate of 20 ml/min. Then, the left atrium was cannulated. Finally, isolated ventilated perfused lung was placed in a temperature-equilibrated housing chamber and freely suspended from a force transducer and recirculation was performed. The whole system was heated from 4ºC to 38ºC. Meanwhile, the flow rate was slowly increased from 20 to 140 ml/min. The left atrial pressure and positive end expiratory pressure (PEEP) were set at 1.5-2.5 cmH_2_O and 2 cmH_2_O respectively. After 20 minutes of steady state period, the perfusate was changed, plasma was added to the perfusate, and the lung was stabilized for additional 20 minutes.

The values of pulmonary artery pressure, left atrial pressure, airway pressure and lung weight were continuously registered using a data acquisition system (Power lab, AD instrument, Australia) connected to pressure and force transducers. All lungs included in the study exhibited criteria similar to our previous studies.^8^^,^^16^

*Composition of Ventilatory Gas and Perfusate*

Four different gas mixtures were employed in this study, namely (i) normoxic normocapnic gas: 21% O_2_, 5.5% CO_2_ balanced with N_2_, (ii) hypoxic normocapnic gas: 3% O_2_, 5.5% CO_2_ balanced with N_2_, (iii) hypoxic gas without CO_2_: 3% O_2_ balanced with N_2_, and (iv) air: 21% O_2_ with 79% N_2_. The perfusate used in this study contained in mM: 120 NaCl, 1.1 K_2_HPO_4_, 1.3 MgPO_4_, 4.3 KCl, 2.4 CaCl_2_, and 13.32 glucose. pH was adjusted to normal physiological range of 7.35-7.40 with NaHCO_3_ in all groups except for the HEPES 1 and HEPES 2 groups (table 1). In the HEPES 1 group, NaHCO_3_ in the perfusate was replaced by HEPES while the lung was ventilated with normoxic and hypoxic gas (contains CO_2_ 5.5%). In the HEPES 2 group, HEPES was used instead of NaHCO_3 _while the lung was ventilated with air and hypoxic gas without CO_2_. pH in the HEPES 1 group was adjusted with NaOH before and during the time course of experiments. pH in the HEPES 2 was adjusted with NaOH and HCL before lung preparation. In the HEPES 2 group, dextran 1% (MW=70,000) and in other experimental groups, plasma (18.08±0.56 ml) were added to the perfusate to preserve oncotic pressure in the system. Osmolarity was adjusted with NaCl in normal range in the HEPES 1 and HEPES2 groups.

**Table 1:** pH, PCO_2_, PO_2_ and HCO_3_- of the perfusate in the experimental groups

	**Control HOX**	**Control NOX**	**DIDS 400**	**DIDS 200**	**HEPES 2**	**HEPES 1**
pH						
Steady state	7.36±0.01	7.32±0.03	7.36±0.01	7.38±0.02	7.41±0.02	6.90±0.00*
Min 0	7.37±0.01	7.38±0.02	7.38±0.01	7.39±0.01	7.41±0.03	7.03±0.05*
Min 30	7.37±0.01	7.36±0.02	7.34±0.01	7.37±0.01	7.42±0.03	7.28±0.06
Min 60	7.41±0.03	7.35±0.02	7.34±0.02	7.37±0.04	7.36±0.02	7.33±0.06
PCO_2_ (mmHg)						
Steady state	33.89±2.01	40.53±2.94	34.30±0.75	36.08±1.74	00.00±0.00*	42.83±1.55
Min 0	34.61±0.88	35.63±1.90	33.00±0.63	35.83±1.43	00.00±0.00*	38.43±2.59
Min 30	33.21±1.26	38.25±1.24	36.27±0.43	37.15±0.33	00.00±0.00*	29.85±2.57
Min 60	33.33±1.33	37.85±1.31	35.80±0.95	35.70±0.96	00.00±0.00*	33.00±0.65
PO_2_ (mmHg)						
Steady state	110.50±1.21	110.00±1.03	102.00±1.55	108.50±2.31	127.33±1.07*	109.50±2.04
Min 0	106.00±0.65	108.67±3.90	100.67±1.58	106.67±0.26	128.33±0.60*	105.50±2.86
Min 30	34.71±1.05 §	109.33±3.80	35.00±0.89 §	34.50±0.60 §	34.67±0.60 §	33.00±0.82 §
Min 60	34.71±0.70 §	111.85±2.92	42.67±4.96 §	35.70±0.96 §	34.33±1.30 §	27.00±0.00 §
HCO_3_^-^ (mmol)						
Steady state	19.50±1.21	20.83±0.21	19.73±0.08	21.50±0.42	0.00±0.00 ∂	0.00±0.00 #
Min 0	20.15±0.68	21.13±0.12	19.93±0.03	21.78±0.32	00.00±0.00*	10.63±0.80*†
Min 30	20.32±0.46	21.23±0.10	19.73±0.15	21.58±0.12	00.00±0.00*	13.98±1.12 †
Min 60	19.73±0.61	21.33±0.11	19.43±0.23	21.40±0.24	00.00±0.00*	18.28±2.08

Values are means±SE in the control hypoxic group (control HOX, n=7), control normoxic group (control NOX, n=4), DIDS (400 μM) treated hypoxic group (DIDS 400, n=3), DIDS (200 μM) treated hypoxic group (DIDS 200, n=5), HEPES 1 (n=4) and HEPES 2(n=4) hypoxic groups. *P<0.05 versus other groups; #P<0.05 versus other groups except HEPES 2 group; †P<0.05 versus HEPES 2 group; §P<0.05 versus control NOX group; ∂P<0.05 versus other groups except HEPES 1 group

*Study Protocol*

Forty minutes after starting the experiment, lungs were ventilated with hypoxic gas for 10 minutes to evaluate lung viability and its responses to alveolar hypoxia as a routine test procedure. Thereafter, the lungs were ventilated with normoxic gas for 20 minutes. Subsequently, the lungs were randomly divided into six groups; one control normoxic group (NOX, n=4) and five hypoxic groups of control (HOX, n=7), DIDS (400 μM, n=3) treated, DIDS (200 μM, n=5) treated, HEPES 2 (n=4), and HEPES 1 (n=4). In the DIDS treated groups, agents were added to the perfusate at 10 minutes before the onset of the hypoxic gas ventilation (after 30 minutes of steady state period). Perfusate samples were taken at min 0, 30, and 60 of experiments in order to measure O_2_, CO_2_,HCO_3_^- ^, pH, and osmolarity at each group.

*Statistics*

Data are the means±SEM. Analysis of variance (ANOVA) with the Student-Newman-Keuls (SNK) post hoc test was used for the comparison of more than two groups. For the comparison of two groups, Student’s t-test was applied. Significance was assumed for P<0.05. 

## Results

Table 1 presents PO_2_, PCO_2_, HCO_3_^-^ and pH of the perfusate during steady state period (30 minutes after lung preparation) and at min 0, 30 and 60 of experiments. The values of PCO_2_, PO_2_, HCO_3_^-^, and pH in the control NOX group remained stable during the time course of experiments. PO_2_ was significantly lower in all hypoxic groups compared with the control NOX group. There was no significant difference between the values of PCO_2_, HCO_3_^-^ and pH during steady state period as well as min 0, 30 and 60 in the control HOX, DIDS 400 µM and DIDS 200 µM groups. Perfusate was HCO_3_^-^ free during the steady state period and at min 0 of the HEPES 1 and HEPES 2 groups. However, ventilation of the lungs with hypoxic gas resulted in the production of HCO_3_^- ^in the HEPES1 group (from CO_2_ into the hypoxic gas) reaching to about normal values at min 60 of experiments. In addition, pH in the HEPES 1 group was significantly lower than other groups during the steady state period and at min 0. However, it was increased gradually at min 30 and 60 of experiments. In the HEPES 2 group, however, the lungs were ventilated with air and hypoxic gas without CO_2_. Therefore, PCO_2_ and HCO_3_^-^ concentration were zero in the perfusate while pH was adjusted at normal range during the time course of experiments.

No significant difference was perceived in the values of LAP, PAW, and LW between the experimental groups during the steady state period. However, the values of PAP in the normoxic group were higher and in the DIDS 200 and HEPES1 groups were lower than other groups (data are not shown). However, no significant alteration was observed between the maximum values of ∆PAP in all experimental groups during short term hypoxic gas ventilation even though PAP tended to be lower with more variation in the HEPES 1 group compared to others (data are not shown). 

**Figure 1:**
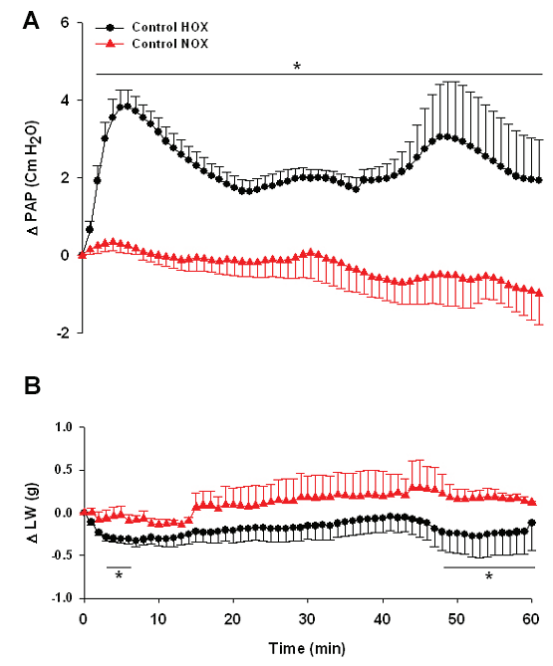
Changes of pulmonary artery pressure (∆PAP) (a), and lung weight (∆LW) (b), during 60 minutes of control HOX (n=7) and control NOX (n=4) groups. Values are means±SE. *P<0.05 versus control NOX group

All values of LAP and PAW were similar and constant in all experimental groups with no change during 60 minutes of experiments (data are not shown). PAP was stable during 60 minutes in the control NOX group. However, in the control HOX group, PAP increased sharply, reaching to maximum values at minutes 5-6. After that, PAP started to go down to the minimum values at min 20 (the end of the acute phase) but it was still higher than the values of PAP in the control NOX group. Then, PAP increased gradually from min 20 to min 60 (sustained phase). All data from min 2 to 60 in the control HOX group were significantly higher than the control NOX group. Concomitant with the increase of PAP in the control HOX group, LW decreased compared with the control NOX group (minutes 4-8, 48-60). The values of LW in the control NOX group were stable during the time course of experiments (figure 1a and 1b).

**Figure 2:**
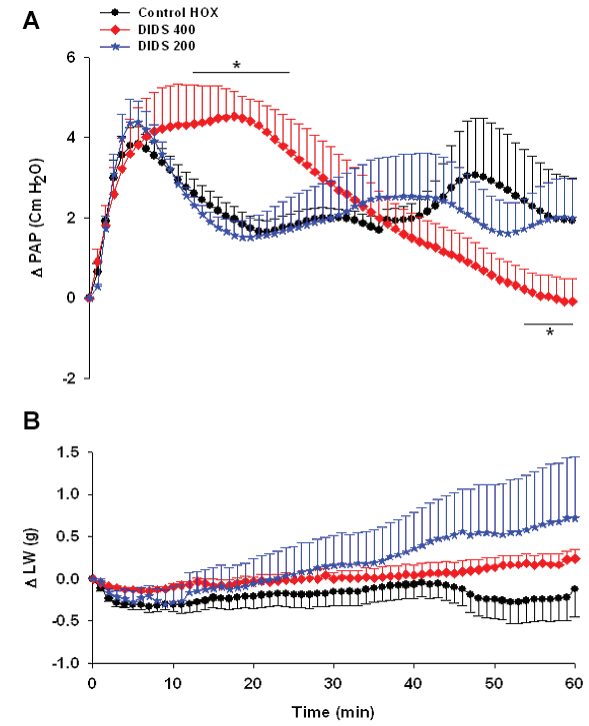
Changes of pulmonary artery pressure (∆PAP) (a), and lung weight (∆LW) (b), in the groups of control HOX (n=7), DIDS 200 (n=5) and DIDS 400 (n=3) during 60 minutes of experiments. Values are means±SE. *P<0.05 versus control HOX group

Administration of DIDS (200 µM) did not change the acute phase as well as the sustained phase of HPV. In the DIDS 400 group, descending part of HPV was prevented and the acute phase extended up to min 24. The values of ∆PAP during min 13-24 in the DIDS group were higher than the control HOX group. However, HPV was decreased gradually during the sustained phase, became significantly lower than the control HOX group during min 56-60. ∆LW was also similar to the control HOX group (figure 2a and 2b).

**Figure 3:**
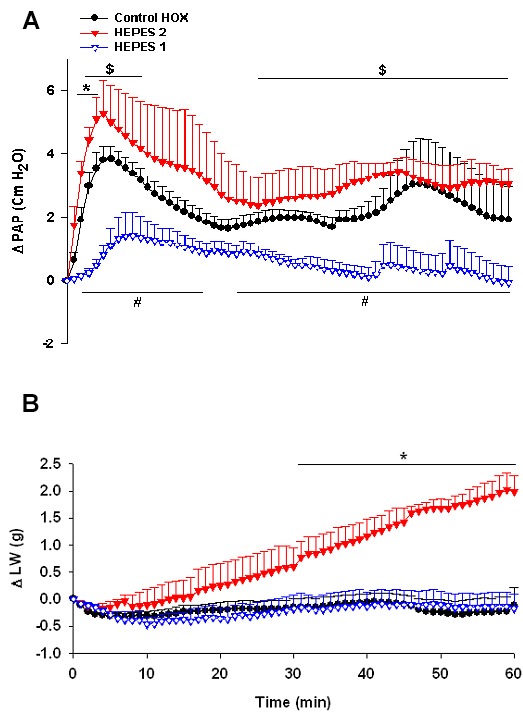
Changes of pulmonary artery pressure (∆PAP) (a), and lung weight (∆LW) (b), in the groups of control HOX (n=7), HEPES 1 (n=4) and HEPES 2 (n=4) during 60 minutes of experiments. Values are means±SE. *P<0.05 in the HEPES 2 group versus control HOX group; #P<0.05 in the HEPES 1 group versus control HOX group; $P<0.05 in the HEPES 2 group versus HEPES1 group

Although ∆PAP in the HEPES 2 group tended to be higher during the acute and sustained phases of HPV, it was only significant during min 2-4 compared with the control HOX group. In contrast, in the HEPES 1 group, both phases of HPV reduced being significant during min 2-19 and 23-60 compared with the control HOX group. Furthermore, the values of ∆PAP in the HEPES 2 group during min 2-10 and min 26-60 were significantly higher than the values of the HEPES 1 group. ∆LW in the HEPES 2 group increased during the time of the experiment became significantly higher during min 31-60 compared with the control HOX group (figure 3a and 3b).

## Discussion

Although, it has been appreciated for more than seventy years that alveolar hypoxia induces hypoxic pulmonary vasoconstriction, the mechanism of action is debatable. No study has yet reported the role of anion exchanger on sustained HPV. Therefore, this study for the first time aimed at addressing the role of anion exchanger during the acute and sustained phases of this physiologic response in the isolated rabbit lung. 

Since pH in all experimental groups, except in the HEPES1 group, were stable during the time course of experiments, any changes in HPV could be unrelated to changes in pH. Basal levels of PAP were different in all groups of experiments. Nevertheless, there was no difference between maximum HPV during short-term hypoxia in all experimental groups. Therefore, basal level of PAP could not influence hypoxic response of pulmonary vasculature and any differences in ∆PAP during 60 minutes of experiments could be independent of basal level of PAP. 

Addition of plasma caused small increase in protein concentration in the perfusate. It was enough to produce oncotic pressure preventing any appearance of pulmonary edema, which could interfere with our data. In the HEPES 2 group, we used dextran instead of plasma to rule out the effect of extracellular HCO_3_^-^ on HPV. Although lung weight in this group increased compared with other groups, but it was still in the endurable range.^21^

No alteration was observed in PAP, LAP, PAW, and LW in the normoxic group, indicating that the time course of experiments did not affect pulmonary vascular tone in our experiments. Biphasic response of HPV in the control hypoxic group was concomitant with the decrease in LW similar to our previous data and others in the isolated rabbit lung.^8^^,^^11^

Although DIDS 200 µM did not affect HPV, DIDS 400 µM extended acute phase and prevented the sustained phase of HPV. DIDS is an anion exchanger and a chloride channel inhibitor. This transporter mediates influx of HCO_3_^−^ in exchange for chloride ion across the cell membrane. Several kinds of anion exchanger are expressed in the lung.^23^^,^^24^ The inhibition of it prevents entry of HCO_3_^-^ into the pulmonary artery smooth muscle cell. Since inhibition of anion exchanger increased the acute phase of HPV, it can be concluded that anion exchanger may modulate acute HPV. However, it is not clear how this anion exchanger modify HPV. It has been shown that DIDS inhibits superoxide release in ischemic reperfusion injury in the isolated perfused lung and rat pulmonary artery.^14^^,^^20^^,^^25^ Consequently, anion exchanger may modify HPV by superoxide release from pulmonary smooth muscle cells.^11^^,^^14^^,^^26^ Prevention of the sustained phase of HPV by DIDS 400 µM is perhaps related to reduction of intracellular HCO_3_^-^ concentration or intracellular pH. These data are comparable to data from others, showing the effects of acetazolamide as a carbonic anhydrase inhibitor on HPV. Acetazolamide decreases HCO_3_^- ^production and intracellular pH,^27^^,^^28^ decreases both the amplitude and kinetics of HPV in the isolated perfused lung,^29^ and prevents the increase of pulmonary artery pressure and pulmonary vascular resistance in conscious dogs ventilating with hypoxic gas.^30^ This drug, increases ventilation-perfusion mismatching and reduces arterial oxygen pressure in conscious dogs.^31^ Furthermore, 90 minutes of hypercapnic acidosis did not potentiate HPV or even weakened HPV transiently in the anesthetized pig, and hypercapnic acidosis did not change HPV during 120 minutes in the isolated perfused rabbit lung.^8^^,^^32^ Therefore, DIDS 400 µM may prevent sustained HPV through the induction of intracellular acidosis. However, more investigations are required to clarify the role of intracellular pH in HPV.

Substitution of HCO_3_^-^ with HEPES in the HEPES 1 group abolished PAP during the acute and sustained phases of HPV. Nozik-Grayck et al., indicated that HCO_3_^-^ free buffer decreased ring tension in the rat pulmonary artery while the lungs were ventilated with hypoxic gas containing CO_2_.^14^ Vankova et al. also showed that, HCO_3_^-^ free medium prevented vasoreactivity of small pulmonary artery during hypercapnia in rat pulmonary artery.^15^ However, none of these studies reported concentration of HCO_3_^-^ in the medium during time course of experiments. In our work, in the HEPES 1 group, pH of the perfusate was lower than other groups because CO_2_ in the hypoxic gas decreased pH. In addition, we measured HCO_3_^- ^concentration in the perfusate during the time course of experiments and found that if the lung is ventilated with normoxic or hypoxic gas (with CO_2_ 5.5%), HCO_3_^-^ concentration is increased for the reaction of CO_2_ and H_2_O. Thus, it was not possible to have HCO_3_^- ^free perfusate buffer. Consequently, in the HEPES 2 group, the lungs were ventilated with air or hypoxic gas without CO_2_. Furthermore, dextran was added to the perfusate instead of plasma (having HCO_3_^-^) in this group. Therefore, there was no HCO_3_^-^ in the HEPES 2 group and the result was more reliable for the evaluation of the role of extracellular HCO_3_^- ^on HPV than the HEPES 1 group. Moreover, we corrected pH precisely before starting the experiment, which remained stable during the time course of experiments in the HEPES 2 group. Therefore, any changes in PAP in this group could not be related to pH. PAP in the HEPES 2 group was a little bit higher than the control hypoxic group, which is again suggesting the role of anion exchanger on the acute phase of HPV.

Overall, since the role of the anion exchanger in the superoxide release has been indicated, and the role of reactive oxygen species in HPV has been proposed, therefore, HCO_3_^- ^free buffer may prevent superoxide release leading to augmentation of HPV especially during the acute phase.^14^^,^^26^

## Conclusion

This study for the first time indicates that, anion exchanger may modulate HPV. Extracellular HCO_3_^- ^may play a role during acute HPV. However, further study is needed to clarify the effect of long-term anion exchanger inhibition on intracellular pH and superoxide release during sustained HPV.
